
JOURNAL INFORMATION
==============================
NLM Title Abbreviation: Atlanta Med Surg J (1884)
NLM Journal ID: 101251623

Atlanta Medical and Surgical Journal (1884)

Publisher: Biomedical Journal Digitization Project

ARTICLE INFORMATION
==============================
PMCID: PMC9201859
Article version: 1
Subjects: Book Reviews, Pamphlets, Exchanges

Magazine Notes

Print publication date: 1899 Feb
Volume: 15
Volume Series: New Ser
Issue: 12
First page: 844
Last page: 844
License: This work is free of known copyright restrictions. For more information, please see PMC Back Issue Digitization.
License URL: https://creativecommons.org/publicdomain/mark/1.0/

==============================

of the papers are well illustrated, which is always an entertaining feature. This association is certainly to be commended for the good work it is doing.

---

MESSRS. LEA BROTHERS & CO. announce the early publication of *The Practice of Obstetrics*. By *American Authors*. They announce that it will be a work of exceptional value. Professor Charles Jewett is the editor.

---

### MAGAZINE NOTES.

THE *Scientific American* continues to be filled with good things. Some of its recent illustrations, amplified by means of the text, have been exceedingly interesting and instructive. When placed upon the table in our reception room it is always read with avidity.

THE *Ladies' Home Journal* for January could scarcely be improved upon. There were some delightful character sketches contained in this issue and the illustrations were exceedingly interesting. This is certainly the journal for the family.

---

### REPRINTS RECEIVED.

Coloring Matter and Ferments. By J. F. Peavy, M.D., Asheville, N. C.

Thrombosis of the Lateral Sinus, Dependent upon Suppurative Otitis Media, with Report of Cases.

The Sequelæ of Middle Ear Suppuration, with Report of Cases. Both by Edward B. Dench, M.D., New York.

Diseases of the Ear as a Specialty. By Emil Amberg, M.D., Detroit, Mich.

The Sixth Annual Report of the Sheppard Asylum, Baltimore, Md.
